# Peroxisomes in the mouse ovary and their alterations during follicular development and oocyte maturation

**DOI:** 10.1007/s00441-025-04025-6

**Published:** 2025-11-23

**Authors:** Claudia Colasante, Eva-Maria Distler, Shan Wang, Phillip Grant, Eveline Baumgart-Vogt

**Affiliations:** 1 Institute for Anatomy and Cell Biology, Division of Medical Cell Biology, Justus Liebig University Giessen, Aulweg 123, 35392 Giessen, Germany; 2 Psychology School, Fresenius University of Applied Sciences, Marienburgstr 6, 60528 Frankfurt am Main, Germany; 3 Department of Biomedical Engineering, The Hong Kong Polytechnic University, Hong Kong, China

**Keywords:** Peroxisome, PPAR, Ovary, Folliculogenesis, Oocyte, PEX14

## Abstract

Peroxisomes are the site of important metabolic pathways as they contain enzymes involved in the β-oxidation of fatty acids, the degradation of reactive oxygen species and the synthesis of ether-lipids and steroid hormone precursors. They are present in virtually all eukaryotic cells, yet they demonstrate considerable heterogeneity of number and protein composition. The presence of peroxisomes and concomitant activity of peroxisomal enzymes was previously documented in ovarian cells and it was suggested that peroxisomal oxidative stress management through catalase plays a pivotal role during oogenesis. In the present article, an exhaustive analysis is presented of the changes in peroxisome abundance during folliculogenesis in mouse oocytes. To this end, a quantitative assessment of the number of peroxisomes was conducted through morphometric analysis in the oocytes in follicular and oestrus cycle stages. Furthermore, a qualitative investigation was undertaken into the distribution of peroxisomal proteins involved in their biogenesis, membrane transport and lipid/hormone and reactive oxygen species metabolism in secondary and tertiary follicles. Given the metabolic connection between peroxisomes and mitochondria, the study was complemented with immunofluorescence stainings for reactive oxygen species and mitochondrial electron transport chain complexes and antioxidative enzymes. Our results confirmed the presence of peroxisomes in all follicular cells, including the oocyte. They further prove that peroxisomal abundance and protein composition are subject to variation depending on follicular and oestrus cycle stage and follicular cell type. Most strikingly, the study provided evidence of the near absence of catalase and of oxidative stress marker 8-oxo-2′-deoxyguanosine in the oocyte.

**Supplementary Information:**

The online version contains supplementary material available at 10.1007/s00441-025-04025-6.

## Introduction

Peroxisomes are ubiquitous, single membrane-bound cell organelles that are present in almost any eukaryotic cell (Rhodin 1955; Baumgart 1997; Baumgart-Vogt et al. 2003; Wanders and Waterham 2006). In ovarian tissue, these organelles were initially visualized and described during an electron microscopic analysis using 3, 3′-diaminobenzidine (DAB)-labelling for the enzyme catalase (Böck 1972). The authors showed that mouse granulosa cells of ripening follicles as well as interstitial cells contained peroxisomes that were much smaller and less numerous compared to the “classical” peroxisomes discovered in liver. They also noticed differences in peroxisome abundance amongst the various ovarian cell types (Böck 1972).

The enzymes localized in peroxisomes catalyze a variety of important metabolic reactions, including the degradation of reactive oxygen species (ROS) (Fransen et al. 2012), the β-oxidation of fatty acids (Hashimoto 1996; Hashimoto et al. 1999), the synthesis of ether-lipids (Wanders 2004), the provision of cholesterol precursors for steroid hormone synthesis (Keller et al. 1986) and the interconversion of 17β-hydroxysteroids (Dieuaide-Noubhani et al. 1996).

Dysfunction or absence of peroxisomes leads to various severe metabolic syndromes (peroxisomal diseases), some of which, like the Zellweger syndrome are lethal. Increasing evidence suggests that functional peroxisomal metabolism is essential for proper development of the female reproductive system and the maintenance of its function: (i) Peroxisomal diseases can cause clitoromegaly in females (Powers et al. 1985; Marjo and Knaap 2006); (ii) Female mice in which the protein responsible for catalyzing the initial step of peroxisomal β-oxidation (ACOX1) has been deleted became sterile (Fan et al. 1996); (iii) Catalase, the peroxisomal marker enzyme that degrades H_2_O_2_ to water and molecular oxygen, is critical for the management of oxidative stress during folliculogenesis, and its abundance is regulated during the oestrus cycle phase (Singh and Pandey 1998); (iv) In mice, the knockout of the peroxisomal enzyme glyceronephosphate O-acyltransferase (GNPAT), which is required for the initial step of plasmalogen biosynthesis (Nagan and Zoeller 2001; Wanders and Waterham 2006), was shown to reduce the number of secondary and tertiary follicles and corpora lutea (Wanders and Waterham 2006) and to lower ovary size and fertility (Rodemer et al. 2003) and (v) A missense mutation in the peroxisomal β-oxidation/steroid interconversion enzyme MFP2 (hydroxysteroid-(17β)-dehydrogenase) resulted in ovarian dysgenesis (Pierce et al. 2010).

How peroxisomal metabolism ensures functional steroidogenesis and how its dysfunction contributes to female infertility has not been yet thoroughly investigated. However, it has become increasingly clear that peroxisomal oxidative stress management through catalase plays a central role during oogenesis (Adrian et al. 1986; Singh and Pandey 1998; Behl and Pandey 2002; Wang et al. 2022). In this paper, we comprehensively characterized peroxisomes in oocytes and somatic cells of mouse ovaries using an optimized indirect immunofluorescence protocol (Grant et al. 2013). To this end, we quantitatively assessed the number of peroxisomes by morphometrical analysis in different types of follicles in different oestrus cycle phases using PEX14-labelling. Moreover, we performed a qualitative analysis of the cellular distribution of peroxisomal proteins involved in their biogenesis, membrane transport and lipid/hormone and ROS metabolism. Because peroxisomes and mitochondria are metabolically coupled during lipid and ROS metabolism (Fransen et al. 2017), we complemented our study with immunofluorescence stainings for ROS, mitochondrial electron transport chain complexes and antioxidative enzymes in different cell types of secondary and tertiary follicles.

## Material and methods

### Animals and ethical statement

For all experiments, female C57BL/6 J mice aged 12–13 weeks were used (Charles River Laboratories, Sulzfeld, Germany). The mice were housed under standard laboratory conditions with constant access to food and water and a 12 h dark-/light-cycle. Anesthesia, euthanisation and dissection of the ovary were approved by the local authorities conforming to the Federal Act on the Protection of Animals (§4 Sect. 3 of the Federal Act on the Protection of Animals-TSchG-) with the university internal classification number JLU-Nr.: 471_M (Project ID: 1016 Peroxisomen).

### Determination of oestrus cycle by histological methods

Vaginal smear tests were routinely performed at the same time each day and stained with Papanicolaou staining to determine the oestrus cycle stage (Supplemental Fig. S1 a–d). To this purpose, the vulva was cleaned with a piece of cotton dampened with saline, and using a pipette tip filled with 10 µl PBS, the vagina was flushed three to five times. The vaginal fluid was thus collected and placed on a glass slide. Thereafter, the slides were stained with Papanicolaou (PAP-test) and examined under light microscope with 10x/40x objective. Four different oestrus cycles were determined as described by Parkening et al. (1982).

Azan staining was further employed to assess the morphology of the vaginal walls and the ovaries at different oestrus cycles stages (Supplemental Fig. S1 e–l). To this purpose, 5 µm tissue sections were first deparaffinized with xylene and then re-hydrated in alcohol in a downstream dilution series (99%, 99%, 96%, 80%, 70%, 50%) 3 min each. The slides were incubated 15 min in azocarmine G solution (0.1% azocarmine G w/v, 1% glacial acetic acid), preheated to 56 °C and then rinsed in dH_2_O. The slides were then transferred to anilinethanol (0.1% v/v) and incubated until only the nuclei appear stained. Samples were then washed for 1 min in 1% v/v glacial acetic acid in ethanol and incubated 2 h in 5% v/v phosphotungstig acid. After rinsing with dH_2_O, slides were stained for 2 h in 1:3 diluted anilin blue-orange-G (0.5% w/v anilinblue, 2% w/v orange-G, 8% v/v glacial acetic acid). Slides were rinsed shortly in dH_2_O followed by sample dehydration and clearance by immersion in 50%, 70% and 80% ethanol (1 min each), 96% ethanol (3 min), and 99% ethanol (2 times 3 min). Finally, the slides were immersed in xylene, allowed to dry and mounted in DEPEX. Light microscopy (Leica DM 750 microscope) was used to capture images. Images were processed and arranged to final figures using Adobe Photoshop CC (Version 20.0.6).

### Dissection and embedding of the mouse ovaries

Animals were anaesthetized using 4% isoflurane and immediately euthanized by cervical dislocation. Thereafter, the mice were perfused retrogradely through the left ventricle with 4% paraformaldehyde, 2% sucrose in PBS (pH 7.4). After fixation, the ovaries were exposed, dissected and placed in a falcon tube containing 4% paraformaldehyde, 2% sucrose in PBS (pH 7.4) for overnight immersion fixation. The next day, the whole organs were paraffin-embedded (Paraplast) using a Leica TP 1020 automated vacuum infiltration tissue processor. For histological stainings, paraffin blocks were cut with a Leica RM2135 rotation microtome to 2-µm-thick sections for immunofluorescence analysis and conventional histological staining respectively.

### Immunofluorescence analysis (IFA)

Sections from ovaries were deparaffinized by immersing them 3 × for 5 min in xylene and subsequently rehydrated in a descending alcohol series consisting of 2 × 99%, 96%, 80%, 70% and 50% ethanol (2 min each step). The tissue sections were incubated with trypsin for 9 min at 37 °C, then placed in 10-mM citrate buffer (pH 6.0) and microwaved for 15 min at 900 W (following the method of Grabenbauer et al. (Grabenbauer et al. 2001). Tissue section blocking was carried out for 2 h at room temperature using a 4% BSA solution prepared in Tris-buffered saline, 0.05% Tween 20 (TBST). Following this, the sections were incubated overnight at room temperature with different primary antibodies (Supplemental Table S1), which were appropriately diluted in 1% BSA in TBS-Tween. The next day, the primary antibody was removed and replaced with the appropriate fluorochrome-conjugated secondary antibody (Supplemental Table S2) diluted in 1% BSA in TBS-Tween. The sections were then incubated for 2 h at room temperature. Nuclei were stained with 1 µmol DAPI for 10 min at room temperature (Molecular Probes/Invitrogen, Carlsbad, CA). Confocal laser scanning microscopy (Zeiss, Laser scanning microscope 167 lsm_710) was used to capture images. The optimal laser intensity for image acquisition was determined for each antibody and maintained throughout the process. Images were processed and arranged to final figures using Adobe Photoshop CC (Version 20.0.6).

To achieve optimal results during immunofluorescence staining, final antibody concentrations were determined using a dilution series. Negative controls were included to ensure the quality and significance of the staining by replacing the primary antibody on the tissue sections with a corresponding volume of PBS. The negative controls were washed separately from the antibody-labelled tissue sections to prevent antibody binding during the washing step. The secondary antibody was used in accordance with the staining protocol. This method made it possible to exclude non-specific staining by the secondary antibodies or tissue autofluorescence.

### Morphometry of peroxisomal density in oocytes and statistical analysis

A total of 45 sections from 7 female C57BL/6 J mice were stained for PEX14 localization (1:2000 dilution) using the method described above. Images (4096 × 4096 Pixel = 190 µm × 190 µm) were taken from 229 follicles with the 63 x objective of a LEICA TCS SP2 CLSM and stored as tagged image files. From the 229 follicles, 32 were primordial, 39 primary, 72 secondary and 86 tertiary follicles. The images were analyzed with ImageJ to calculate the area density of peroxisomes.

Prior to morphometric analysis, the scale for the images was set (4096 Pixel = 190 µm) to allow automatic calculation of the area of the region of interest (ROI). Then using ImageJ, the area of the oocyte (ROI) was marked with a circle, excluding the region of the nucleus when visible (Supplemental Fig. S2). The image was then converted into an 8-bit image, and the required threshold value was determined individually for each antibody by comparing the black and white binary image to the original, coloured image. The threshold value was selected at which both images displayed identical particle (“peroxisome”, “peroxisomal particles”) size, distribution and number. The so determined threshold value was applied to each image from one antibody series to permit standardized, automatic identification of “peroxisomal particles”. After adjusting the threshold, the number of “peroxisomal particles” was counted and the percentage of the ROI they occupied was automatically calculated using the “analyze particles” function in ImageJ. To this purpose, all particles with an area of 0.01–0.1 µm^2^ were taken in consideration.

The individual section thickness of the PEX14-stained area was analyzed by taking separate images scanned with the XZ-mode (Supplemental Fig. S3). The section thickness was measured at five different regions of interest (ROI), and the mean of the five values was calculated, which was set as “verified section thickness”.

All statistical analyses were performed using the Statistical Package for the Social Sciences (SPSS, version 15.0.1). The effects of follicular development and of oestrus cycle on peroxisomal density were analyzed using analysis of variance (ANOVA). To calculate the area density of peroxisomes within any given follicle at any given stage of the oestrus cycle, the area of peroxisomes (as indicated by positive PEX14-staining) was divided by the respective total measurement-area. Additionally, it was considered that a number of factors other than the group-variables “follicle stage” and “oestrus cycle” would explain significant variance in the dependent variable (DV) “peroxisomal density” and, thus, may mask the effects of the group-variables or—at least—result in a considerable loss of statistical power (Miller and Chapman 2001). These covariates were (1) the thickness of any given individual section, (2) the idiosyncrasy of each animal as well as (3) the properties of an individual section (e.g. due to inclination or distance to follicle-center of the section). To remove variance from the DV caused by the aforementioned covariates and not lose degrees of freedom (and, thereby, statistical power), the hierarchical regression method suggested by Cohen et al. (1983) was used: First, only the covariates were entered into the statistical model and (standardized) residuals (DVres) were saved, which were now free of variance explained by the covariates. Subsequent analyses of variance were performed on these (standardized) residuals. In other words: Since, e.g. thickness of any given section would impact the signal-density of PEX14-staining (possibly more so than the follicle stage or oestrus cycle) and lead to “noise” within the DV, removing variance from the DV explained by these confounding variables through hierarchical regression analyses, thus, removes this “noise”, as is the resulting residuals (DVres) are now free of variance explained by the “noisemaking” covariates. This approach, however, has the drawback that DVres is not an absolute indicator of peroxisomal density, but only a relative one (with an overall mean of 0 and a standard deviation of 1).

## Results

### PEX14 is the ideal marker for the detection of peroxisomes in all ovarian cell types during follicular development in different oestrus cycle stages

**Fig. 1:**
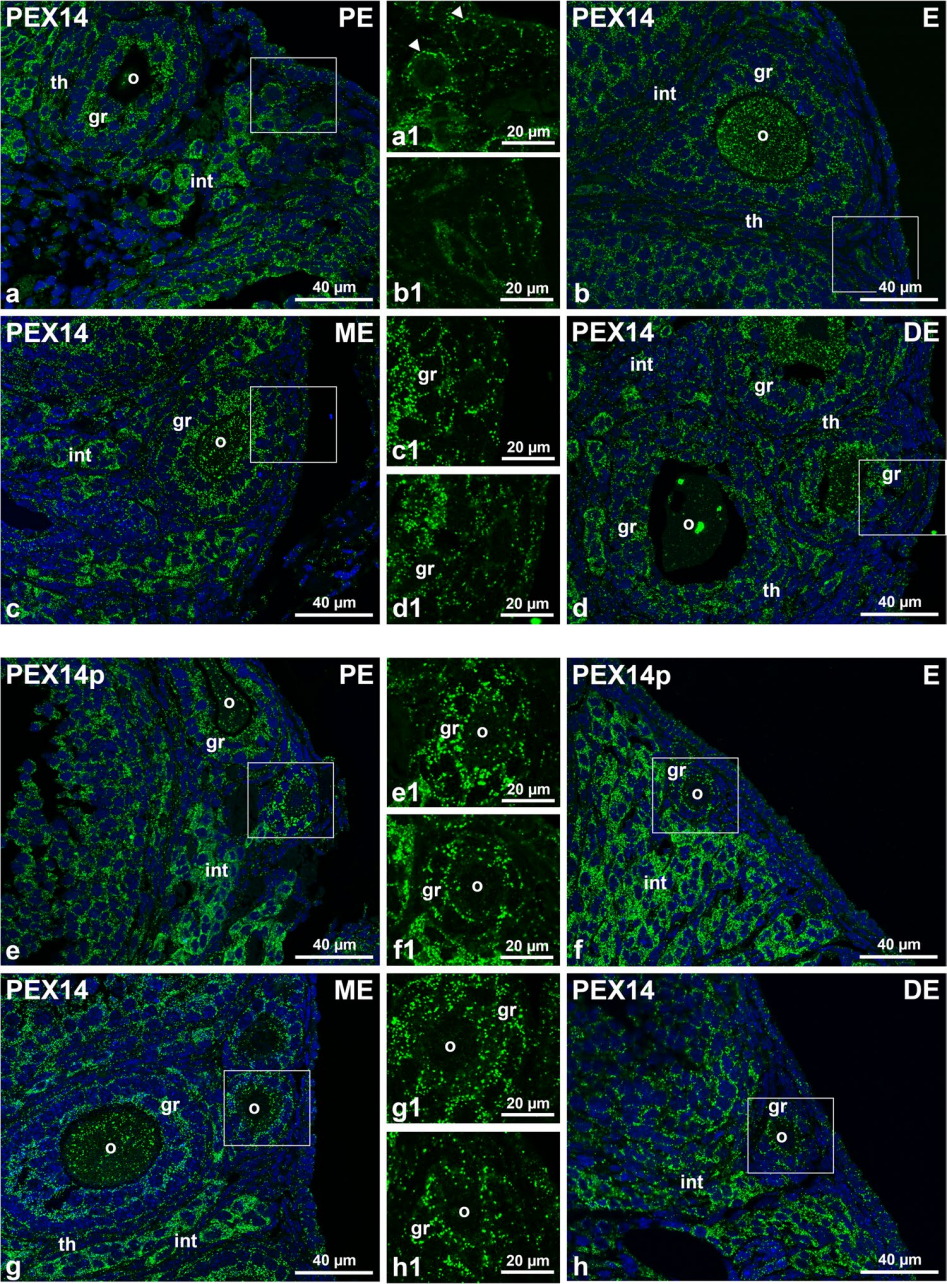
Immunofluorescence analysis of follicles in four different phases of the oestrus cycle. **a**–**h** PEX14-stained primordial (**a**, **b** and **c** and **d**) and primary follicles (**e**, **f**, **g** and **h**). Nuclei were counterstained with DAPI and are shown in blue. Higher magnifications of the region within the squares in **a**–**h** (a1–h1). Abbr.: PE, proestrus; E, oestrus; ME, metestrus; DE, dioestrus; o, oocyte; gr, granulosa cells; th, theca cells; int: interstitial hormone producing cells

**Fig. 2:**
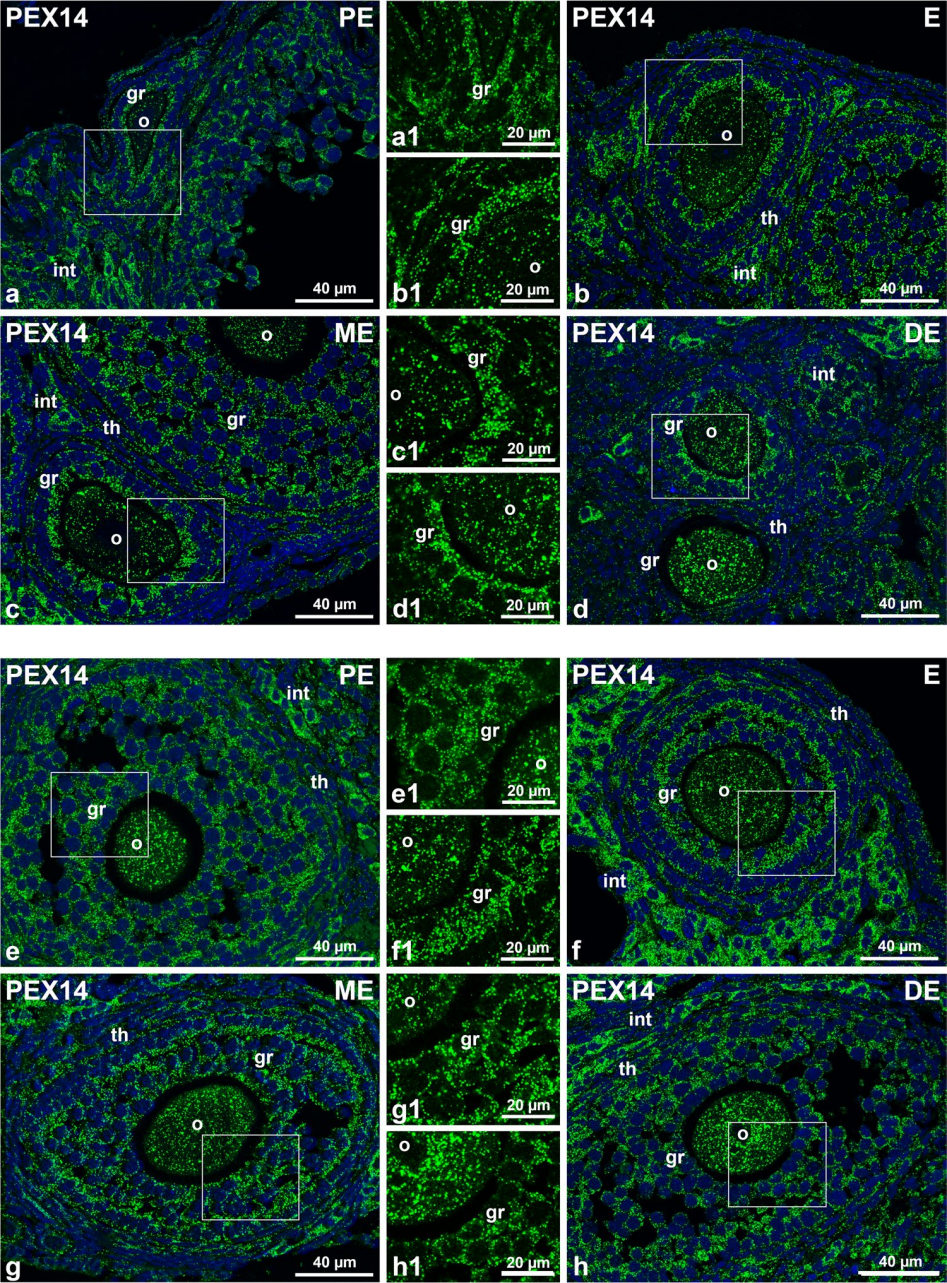
Immunofluorescence analysis of PEX14 in secondary and tertiary follicles in four different phases of the oestrus cycle.** a**–**h** PEX14-stained secondary (**a**, **b**, **c** and **d**) and tertiary follicles (**e**, **f**, **g** and **h**). Nuclei were counterstained with DAPI and shown in blue. Higher magnification of the squares indicated in **a**–**h** (a1–h1). Abbr.: PE, proestrus; E, oestrus; ME, metestrus; DE, dioestrus; o, oocyte; gr, granulosa cells; th, theca cells; int: interstitial hormone producing cells

The coordination of the peroxisomal biogenesis and replication is facilitated by a family of soluble and membrane-associated proteins (peroxins, PEX) that are responsible for peroxisomal membrane assembly, matrix enzyme import and fission. The peroxin PEX14, an integral peroxisomal membrane protein involved in matrix protein import, is ubiquitously expressed in all cell types containing peroxisomes. Since this peroxin is present in detectable amounts on individual peroxisomes, it is a well-suited marker for the morphometric analysis of the peroxisomal population and its alterations (Grant et al. 2013; Colasante et al. 2017). After determining the mouse oestrus cycle stage using PAP-test and Azan stainings of the ovary and the vagina (Supplemental Fig. S1), we used PEX14 immunofluorescence analyses to assess the changes in peroxisome number occurring during folliculogenesis in different ovarian cell types. As expected, PEX14 was readily detectable in all investigated follicular stages and oestrus cycle phases (Figs. 1 and 2). The clearly labelled peroxisomes were individually discernible in the oocyte, the granulosa cells, the theca cells and the interstitial cells (Figs. 1 and 2). The images also revealed that in all follicular cells and in all oestrus cycle stages, peroxisomes were more abundant in secondary and tertiary follicles (Fig. 2) when compared to primordial and primary follicles (Fig. 1). In the cells of primordial follicles (Fig. 1 a1, arrows), peroxisomes frequently appeared positioned like “beads on a string”. In early secondary follicles, PEX14-labelled peroxisomes were found in particularly high concentrations in the inner layer of granulosa cells, which are directly adjacent to the oocyte (Fig. 2a–d). In the tertiary follicle, this characteristic distribution was no longer observable. Instead, the PEX14-stained peroxisomes were found to be distributed equally throughout the layers of granulosa cells except the most external ones in which most peroxisomes were facing toward the apical side (inner side of the follicle) (Fig. 2e–h).

### Quantitative analysis of PEX14 abundance in oocytes during follicle development and oestrus phases

**Fig. 3:**
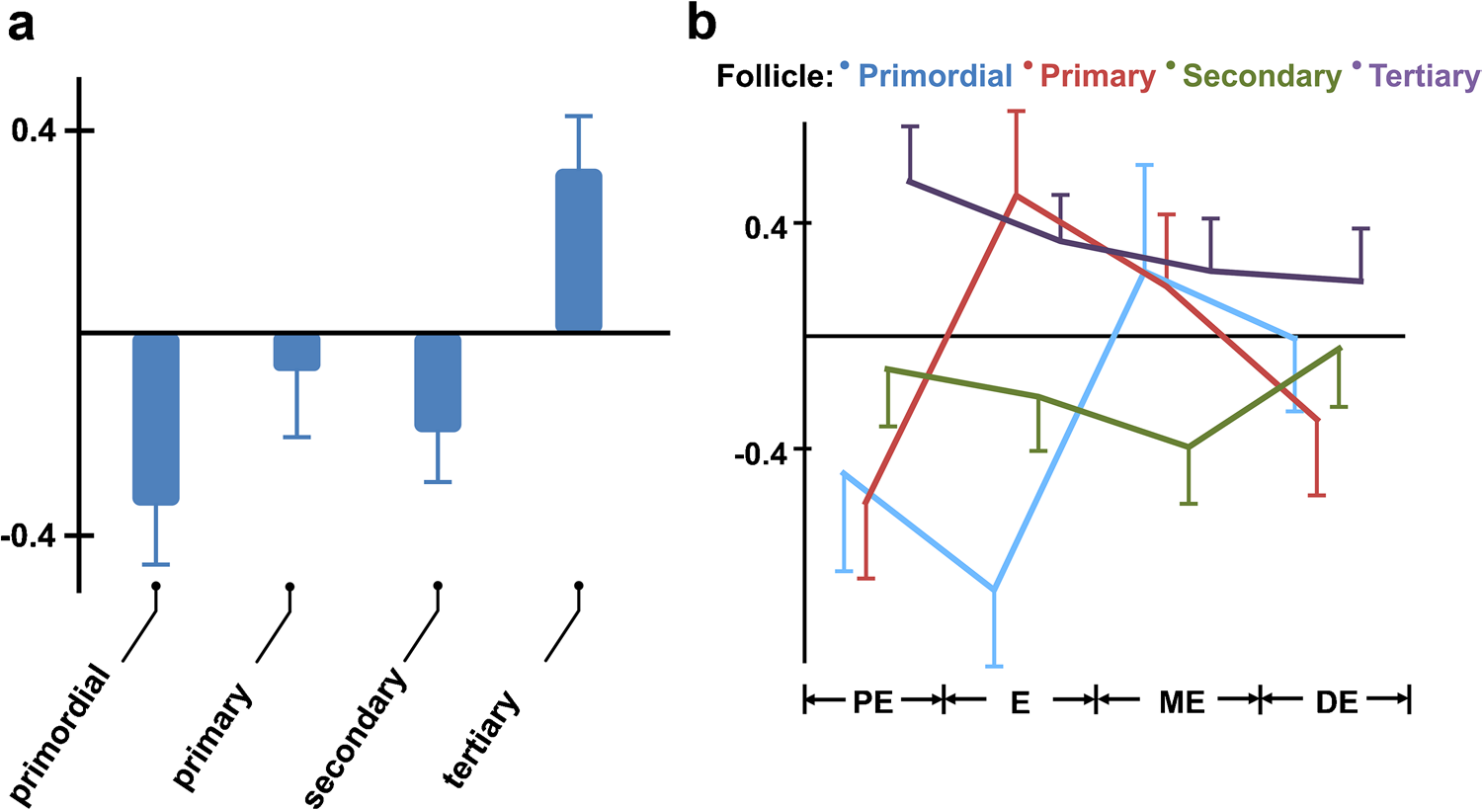
Relative changes in peroxisomal abundance in oocytes during the oestrus cycle.** a** Significant main effect of follicle stage on peroxisomal density (*p* < 0.001). On the graph, standardized residuals with standard errors of the means are shown. **b** Significant interaction between follicular development and oestrus cycle on peroxisomal density (*p* = 0.002). On the graph, standardized residuals of peroxisomal densities are plotted with standard errors of the means. Statistical analysis was performed using SSPS and one-way ANOVA (**a**) or two-way ANOVA (**b**). Abbr.: PE, proestrus; E, oestrus; ME, metestrus; DE, diestrus

After determining the exact oestrus cycle phase by vaginal cell smears and characterization of the vaginal and ovarian histology (Supplemental Fig. S1), we quantified peroxisomal density in oocytes of different follicular stages and oestrus phases by morphometric analysis (Fig. 3). One-way ANOVA of peroxisomal density showed no effect of oestrus cycle (F_3, 225_ = 0.005, *p* = 1.0), while it showed a significant main effect of follicle stage (F_3, 225_ = 7.03, *p* < 0.001). This result was confirmed using post hoc analyses that demonstrated significantly elevated peroxisomal density in tertiary follicles compared to primordial, primary and secondary follicles (*p* < 0.001), respectively. All other individual comparisons were not significant, and an ex post facto ANOVA showed that there was no main effect of follicle stage on peroxisomal density when tertiary follicles were excluded from the analysis (F_2, 140_ = 0.977, *p* = 0.379) (Fig. 3a).

Using a two-way 4 × 4 ANOVA, we further could prove a significant interaction between follicle stage and oestrus cycle on peroxisomal density (F_6, 131_ = 3.65, *p* = 0.002) (Fig. 3b): While the influence of the oestrus cycle was weakest in secondary and strongest in primordial and primary follicles, each stage of follicular development reached its individual peak in peroxisomal density during a different phase of the oestrus cycle: For tertiary follicles, this was the proestrus, for primary follicles the oestrus and for primordial follicles the metestrus, while secondary follicles showed the (relatively) highest peroxisomal density during the dioestrus (Fig. 3b).

### Peroxisomal biogenesis proteins are highly abundant in the mouse ovary

We next wanted to characterize the distribution of different peroxins in secondary and tertiary follicles in more detail.

PEX3 and PEX19 are peroxins that cooperate during early peroxisomal membrane biogenesis and that are crucial for de novo peroxisomal membrane formation by importing peroxisomal membrane proteins (PMPs) into the nascent peroxisome (Eckert and Erdmann 2003; Colasante et al. 2017). While PEX3 is usually located at the peroxisomal membrane, PEX19, a shuttle protein for PMPs, can be found, depending on the cell type, either in the cytoplasm or associated with the peroxisomal membrane through PEX3-docking (Götte et al. 1998; Liu et al. 2016; Colasante et al. 2017).

**Fig. 4:**
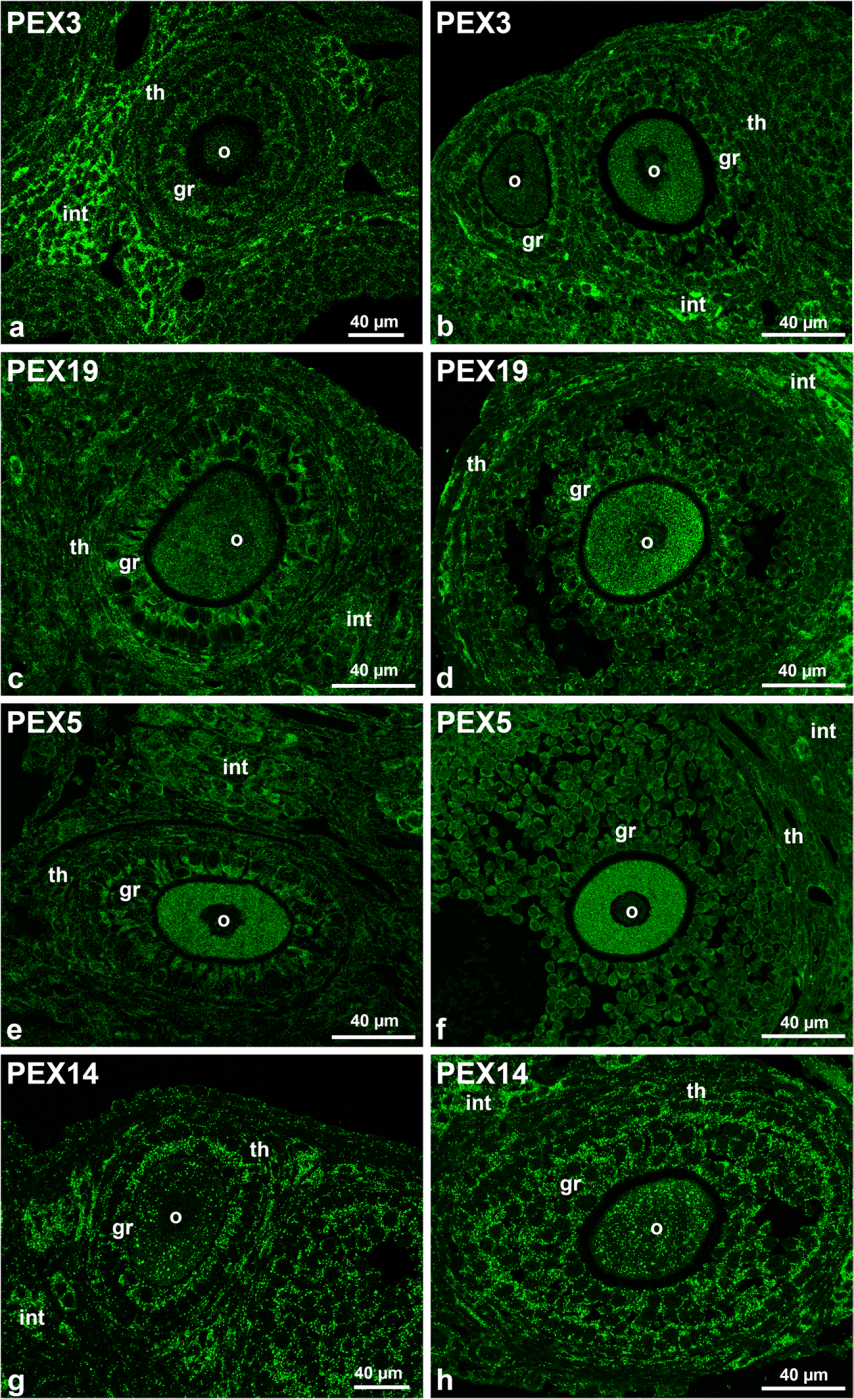
Immunofluorescence analysis of peroxisomal biogenesis proteins in secondary and tertiary follicles.** a**–**h** For the immunofluorescence analysis, antibodies against the peroxisomal proteins PEX3 (**a** and **b**), PEX19 (**c** and **d**), PEX5 (**e** and **f**) and PEX14 (**g** and **h**) were used. Secondary follicles are presented in **a**, **c**, **e** and **g** while tertiary follicles in **b**, **d**, **f** and **h**. Abbr.: o, oocyte; gr, granulosa cells; th, theca cells; int: interstitial hormone producing cells

Our immunofluorescence analysis revealed many PEX3-stained peroxisomes in the interstitial cells that surround the follicle (Fig. 4a and b). The number of detected PEX3-stained peroxisomes was much lower in the theca cells and granulosa cells in both secondary and tertiary follicles (Fig. 4a and b). In the oocytes of secondary follicles, only very few PEX3-stained peroxisomes were observed, while in the tertiary follicle, their number was drastically increased (Fig. 4a and b).

The intracellular distribution of PEX19 slightly differed from the one observed for PEX3 by displaying a combined cytoplasmic and peroxisomal labelling (Fig. 4c and d). In secondary and tertiary follicles, PEX19-stained peroxisomes were observed in granulosa cells and theca cells (Fig. 4c and d). Like in the PEX3-stained follicles, the secondary oocytes stained with PEX19 revealed only few peroxisomes. Their number and size however increased in the tertiary oocytes (Fig. 4c and d).

PEX5 is the cytoplasmic shuttling receptor for the import of most peroxisomal matrix proteins (Dammai and Subramani 2001). PEX5 was extremely high abundant in the cytoplasm of the oocyte in comparison to the one of other cell types and more abundant in tertiary compared to secondary oocytes (Fig. 4e and f). Additionally, to the cytoplasmic staining, PEX5-positive peroxisomes were visible in the oocytes and the granulosa cells (Fig. 4e and f).

For direct comparison with the localization of PEX3 and of the two shuttling receptors (PEX19 and PEX5), similar regions containing secondary and tertiary follicles are presented using PEX14 staining (Fig. 4g and h). In contrast to the other 3 biogenesis markers, PEX14 clearly stains individual peroxisomes in theca and granulosa cells as well as in the oocyte, with no evident cytoplasmic staining (Fig. 4g and h).

Noteworthy, like we observed for PEX14, in all secondary follicles, PEX3, PEX19 and PEX5 were most abundant in the inner layer of the granulosa cells sheet that faces towards the oocyte (Fig. 4a, c, e and g). This was not observable in the large tertiary follicles (Fig. 4b, d, f and h).

### Peroxisomal metabolic enzymes are heterogeneously distributed in distinct cell types of the mouse ovary

Similarly to the above-described distribution of peroxins in distinct cells of the follicles, we have investigated how different peroxisomal metabolic proteins are distributed throughout the different cell types of these follicles.

**Fig. 5:**
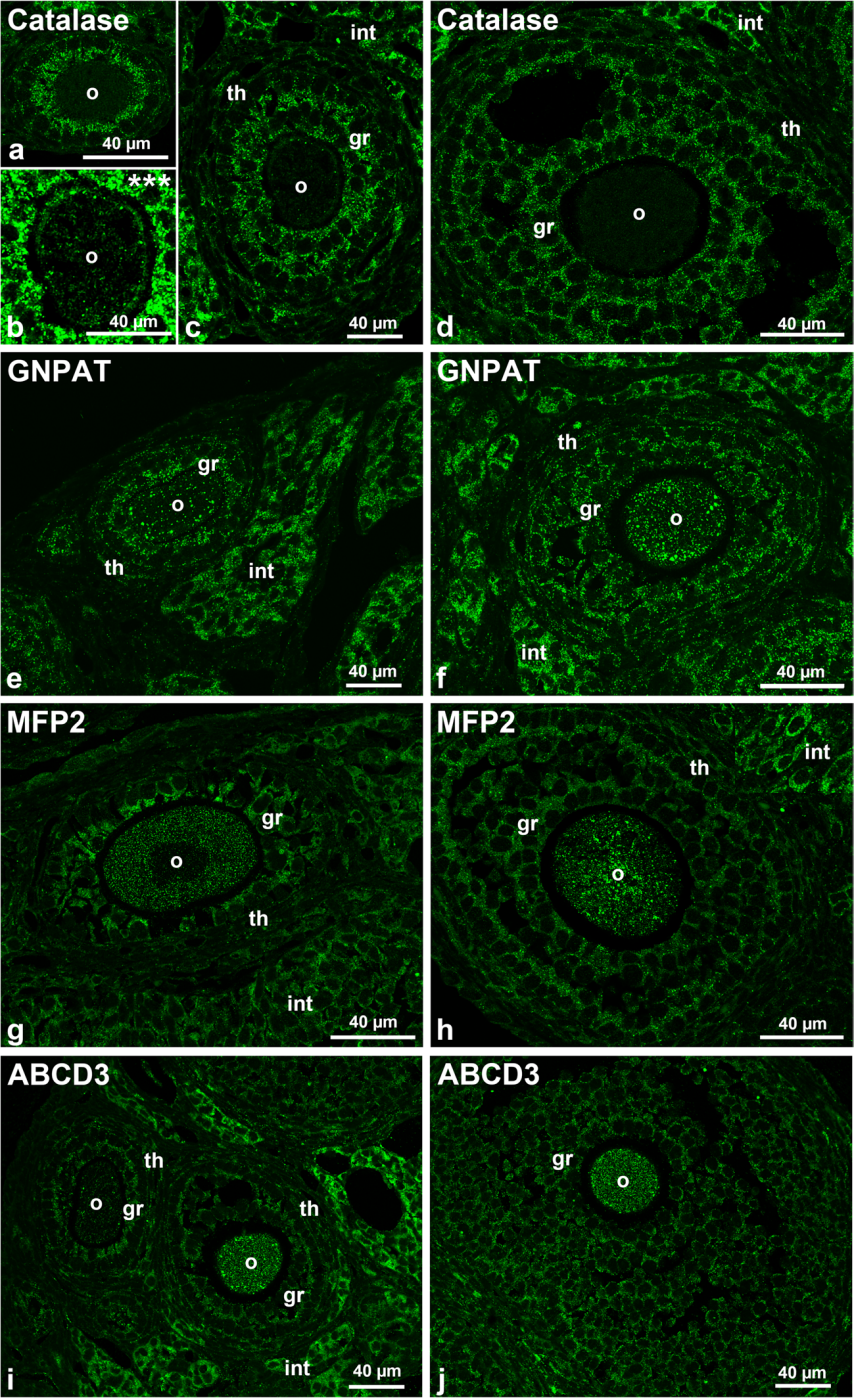
Immunofluorescence analysis of peroxisomal metabolic enzymes and the membrane transporter ABCD3 in secondary and tertiary follicles.** a**–**j** For the immunofluorescence analysis, antibodies against the peroxisomal proteins catalase (**a**, **b**, **c** and **d**), GNPAT (**e** and **f**), MFP2 (**g** and **h**) and ABCD3 (**l** and **j**) were used. Secondary follicles are presented in **a**–**c**, **e**, **g** and **i** while tertiary follicles in **d**, **f**, **h** and **j**. **b** Higher magnification with higher exposure time of the oocyte. Abbr.: o, oocyte; gr, granulosa cells; th, theca cells; int: interstitial hormone producing cells

Catalase is the classic peroxisomal marker enzyme, which has been commonly used for the detection of peroxisomes in a variety of tissues (Fahimi and Baumgart 1999). We found that in secondary and tertiary follicles, catalase-stained peroxisomes were highly abundant in granulosa cells as well as in interstitial cells, while in the oocytes, they were scarcely detectable even with the best condition used for the visualization of the enzyme in the other ovarian cell types (Fig. 5a, c and d). Despite this, we decided against increasing the concentration of antibody to detect catalase in the oocyte to avoid background staining. Instead, we used higher magnification and much longer exposure times and clearly detected catalase in small peroxisomes inside the oocyte cytoplasm as well as in larger ones at the periphery of the oocyte in close association with the zona pellucida (Fig. 5b). Catalase-stained peroxisomes were also low abundant but still detectable in the theca cells. Like we observed for the peroxins in secondary follicles, catalase was strongly stained in the inner granulosa layer directly facing the oocyte.

In contrast to catalase, peroxisomes could be easily visualized in the oocytes of secondary and tertiary follicles using two other antibodies against peroxisomal matrix enzymes (GNPAT, which is involved in ether lipid biosynthesis, and MFP2, which is a peroxisomal β-oxidation enzyme), as well as the fatty acid transporter ABCD3 (Fig. 5e–j). In contrast to GNPAT, MFP2 and ABCD3 were less abundant in the granulosa cells compared to the oocyte (Fig. 5e–j). All three protein were less abundant in the theca cells but very high abundant in the interstitial cells and their amount increased during maturation from secondary to tertiary follicles (Fig. 5e–j). Table 1 summarizes the distribution of the different antibody-labelled peroxisomes in the various ovarian cell-types.

**Table 1:** Distribution of differentially stained peroxisomes in the various ovarian cell-types

	PEX14		PEX3		PEX19		PEX5		catalase		GNPAT		MFP2		ABCD3	
	Comments	Staining	Comments	Staining	Comments	Staining	Comments	Staining	Comments	Staining	Comments	Staining	Comments	Staining	Comments	Staining
Interstitial cells	Large po	+ + + +	Many po	+ + + +		+ + + +	cyt	+ + +		+ + + +		+ + + + +		+ + +		+ + + +
Pregranulosa cells primordial foll	Few, large po	+									Large po	+ + +				
Cuboid granulosa cells primary foll	large po	+ + +														
Granulosa cells secondary foll	Large po	+ + + +		+ +	cyt and large po; inner GC more intense	+/+ +	po; internal layer stronger and apically stained	+ +/+ + +	po apically stained	+ + +	Large po	+ + +		+ + +	Inner GC stronger	+/+ +
Granulosa cells tertiary foll	Large po	+ + + +		+ +	cyt and large po; corona radiata stronger than marginal GCs	+ +/+ + +	po and cyt	+ +/+ + +	po apically stained	+ + +	Large po	+ + + +	Some large po	+ +		+ +
Oocyte primordial foll	Barely visible staining	-									Barely visible staining	-				
Oocyte primary foll	Very small po	+								+		-				+ +
Oocyte secondary foll	Few, large po	+ + +	Large po	+		+	cyt	+ + + +	Large po at the border	+ +	Few very large po	+ + +	Many, small po	+ +	Many po, some large po	+ + +
Oocyte tertiary foll	Large po	+ + + +		+ + +	Large po and cyt	+ + +	cyt	+ + + + +		+	Very large po	+ + + +	Many, large po	+ + + +	Many po, some large po	+ + +
Theca cells sec	Large po	+ + +	Less po	+		+ +	cyt	+		+		+ +		+		+
Theca cells tert	Large po	+ + +	Less po	+		+ + +	cyt; theca int. stronger	+ +/+ + +	Some large po	+ +	Large po	+ +		+		+/+ +

### Peroxisomal proteins catalase and PEX5 are most abundant in large lutein cells

During the formation of the corpus luteum following ovulation, the granulosa cells differentiate into granulosa (large) lutein cells and the theca interna cells develop into theca (small) lutein cells. We analyzed the distribution of peroxisomal proteins within these two cell types in the metestrus phase using antibodies that allowed the best interpretation of the peroxisomal alterations during folliculogenesis, namely those against PEX14, PEX5, catalase and ABCD3.

**Fig. 6:**
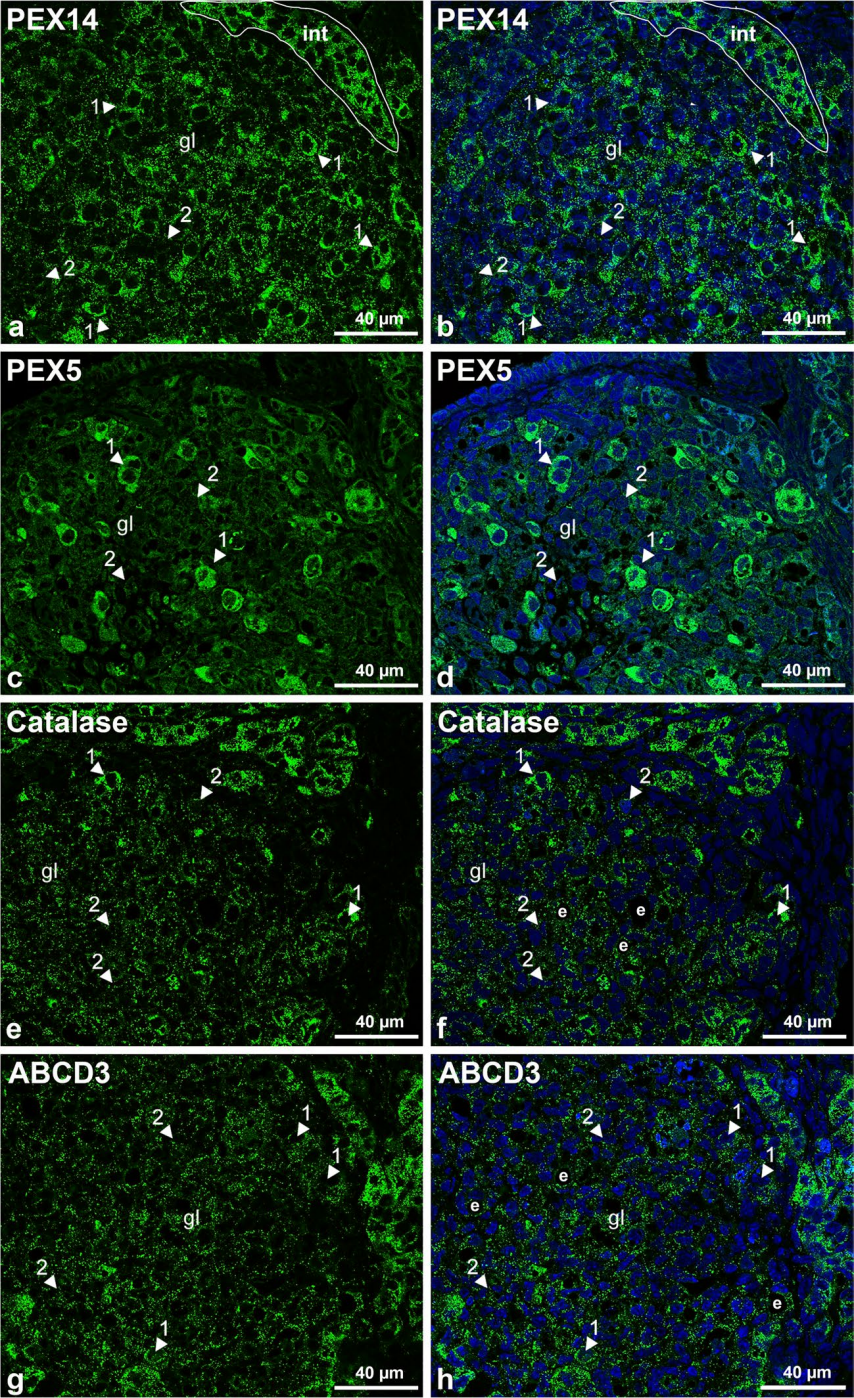
Immunofluorescence analysis of peroxisomal enzymes in the corpus luteum of mouse ovaries.** a**–**h** Sections of ovaries in the metestrus phase were incubated with antibodies against PEX14 (**a** and **b**), PEX5 (**c** and **d**), catalase (**e** and **f**) and ABCD3 (**g** and **h**). The arrows indicate large (1) and small (2) lutein cells. Nuclei were counterstained with DAPI and are shown in blue. Abbr.: lg, luteal gland; int: interstitial hormone producing cells; e: endothelium

In the large lutein cells derived from granulosa cells that are distributed throughout the corpus luteum, PEX14 staining was readily detected (Fig. 6a and b, arrow 1). The intensity of the staining of many of the large lutein cells was comparable to the one observed in the smaller and more compact interstitial cell areas. In the small lutein cells, derived from the theca interna cells, the PEX14 staining was less intense (Fig. 6a and b, arrow 2). The cytoplasmic staining for PEX5 was even more heterogenous in the two distinct lutein cell types, with the large ones exhibiting an extremely strong staining (Fig. 6c and d, arrows 1 and 2). The labelling intensity and distribution for catalase differed in comparison to the previously observed stainings for PEX14 and PEX5. Catalase was more homogeneously distributed amongst small and large lutein cells in the central part of the corpus luteum. The very intensively catalase-stained peroxisomes in the central part of the corpus luteum probably belong to endothelial cells of capillaries (Fig. 6f). The distribution of the lipid transporter ABCD3 within the granulosa and the interstitial cells was very similar to the one observed for catalase (Fig. 6g and h).

Apart from the main follicles, from which the oocytes are released to be fertilized, the other follicles undergo remodeling and decrease in size to increase the interstitial cell hormone production (Rolaki et al. 2005). In the remodeled follicles with degenerating oocytes, we observed that peroxisomes were arranged in “clusters” as detected using PEX13, PEX14 and GNPAT antibodies (Supplemental Fig. S4). Some of the aggregated peroxisomes formed ring-like structures (Supplemental Fig. S4, insets), which resemble peroxisomal alterations and cluster arrangement in the cytoplasm of elongated spermatids and residual bodies during spermiogenesis previously described by our group (Nenicu et al. 2007). In the surrounding granulosa and theca cells of these degenerating oocytes, the typical peroxisomal staining pattern was observed using the antibodies against PEX14 and GNPAT (Supplemental Fig. S4).

### Oxidative stress marker 8-oxo-2′-deoxyguanosine and anti-oxidative enzyme SOD2 are nearly absent in the oocyte

**Fig. 7:**
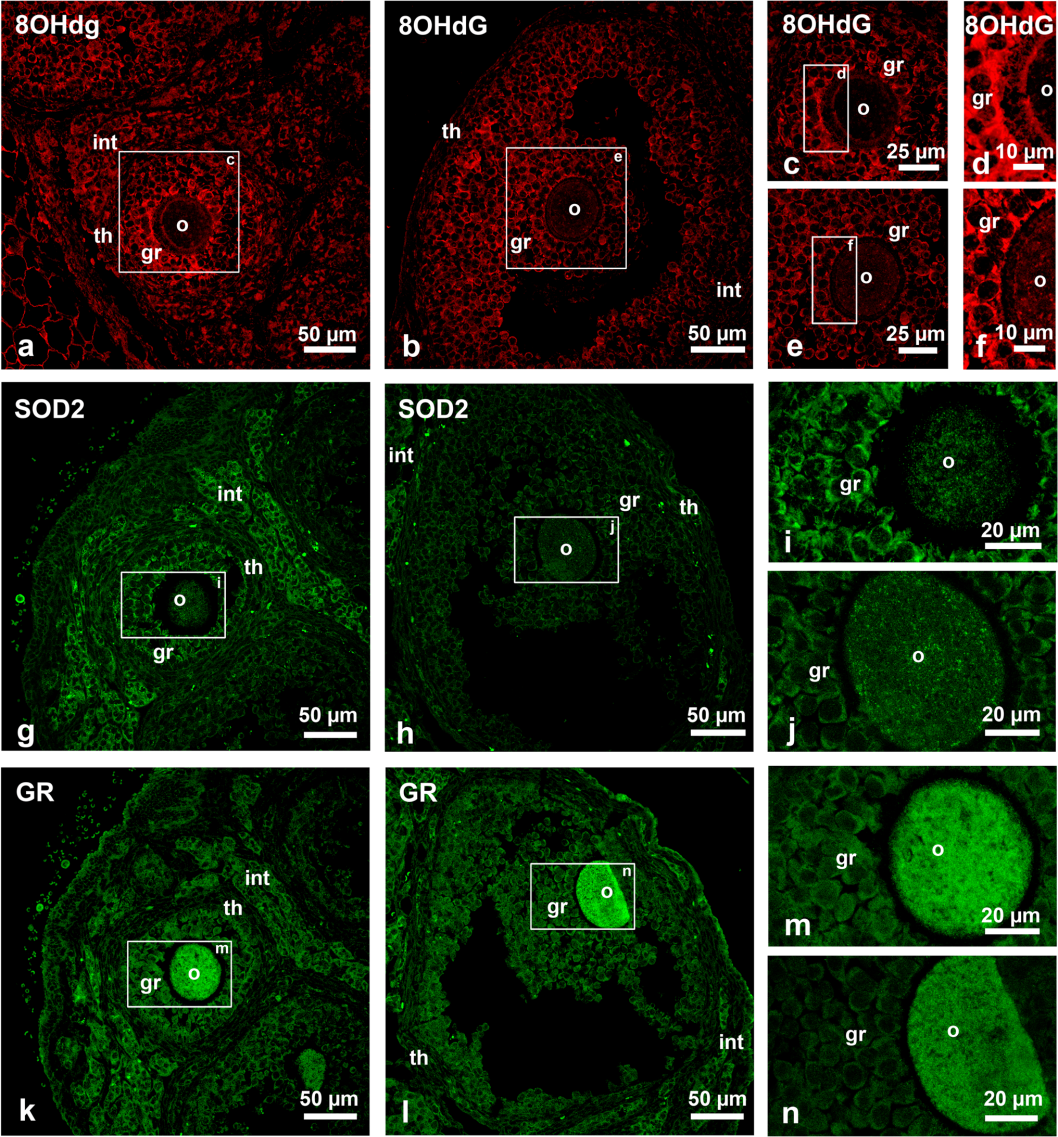
Immunofluorescence analysis of ROS and antioxidative enzymes in secondary and tertiary follicles. **a**–**h** Ovary sections incubated with antibodies against 8OHdG (**a**–**f**), SOD2 (**g**–**j**) and GR (**k**–**n**) in secondary (**a**, **c**, **d**, **g**, **i**, **k** and **m**) and tertiary follicles (**b**, **e**, **f**, **h**, **j**, **l** and **n**). Small images in the third and fourth image columns represent magnification of the overview images of the secondary and tertiary follicles as indicated by the white squares labelled with the appropriate letter. Abbr.: o, oocyte; gr, granulosa cells; th, theca cells; int: interstitial hormone producing cells

The near absence of catalase in the oocyte made us speculate about the overall distribution of ROS in secondary and tertiary follicles. To determine the localization of oxidative stress, we detected the distribution of 8-oxo-2′-deoxyguanosine (8OHdG), a known biomarker for oxidative stress and indicative for the presence of DNA damage. Our results show that 8OHdG preferentially accumulated in the granulosa cells and only partially in the theca cells (Fig. 7a and b). The oocyte demonstrated a peculiar 8OHdG distribution, which was particularly evident in the secondary follicles (Fig. 7a): In both follicle stages, 8OHdG was barely detected in the oocyte; however, at the periphery of the oocyte adjacent to the zona pellucida, an 8OHdG ring was observed (Fig. 7a–c and e). Strong magnification of this part showed that 8OHdG was found also in “cellular bridges” crossing the zona pellucida and reaching from the granulosa cells (Fig. 7d and f).

Superoxide dismutase (SOD2) is a mitochondrial antioxidant enzyme that catalyzes the dismutation of superoxide anions into H_2_O_2_. When we stained the mouse ovary with an antibody against this protein, we found that the secondary follicles where much more intensively labelled than tertiary follicles (Fig. 7g–j). In secondary follicles, we noticed a pronounced SOD2 fluorescence in patchy spots in the granulosa cells and the interstitial cells (Fig. 7g and i). Here, the oocyte contained a diffuse, coarsely granular staining with individual brighter spots (Fig. 7i). Also, the cells of the theca interna were barely labelled, while the interstitial cells displayed strong labelling (Fig. 7g). In contrast to the secondary follicle, in all cells of the tertiary follicles, the staining for SOD2 was very weak (Fig. 7h). Only individual interstitial cells were marked more strongly (Fig. 7h). Within the oocytes, brighter SOD2 stained spots could be visualized (Fig. 7j). Glutathione reductase (GR) is another important enzyme of the oxidative defense system. In contrast to SOD2, GR was particularly strong in the oocytes but not in granulosa cells (Fig. 7m and n). The staining intensity for GR was similar in tertiary and secondary follicles (Fig. 7k and l). Like for SOD2, the staining was almost absent in the theca cells but more prominent in the interstitial cells (Fig. 7k and l).

### Complexes of the ETC are relatively low abundant in oocytes compared to granulosa cells

**Fig. 8:**
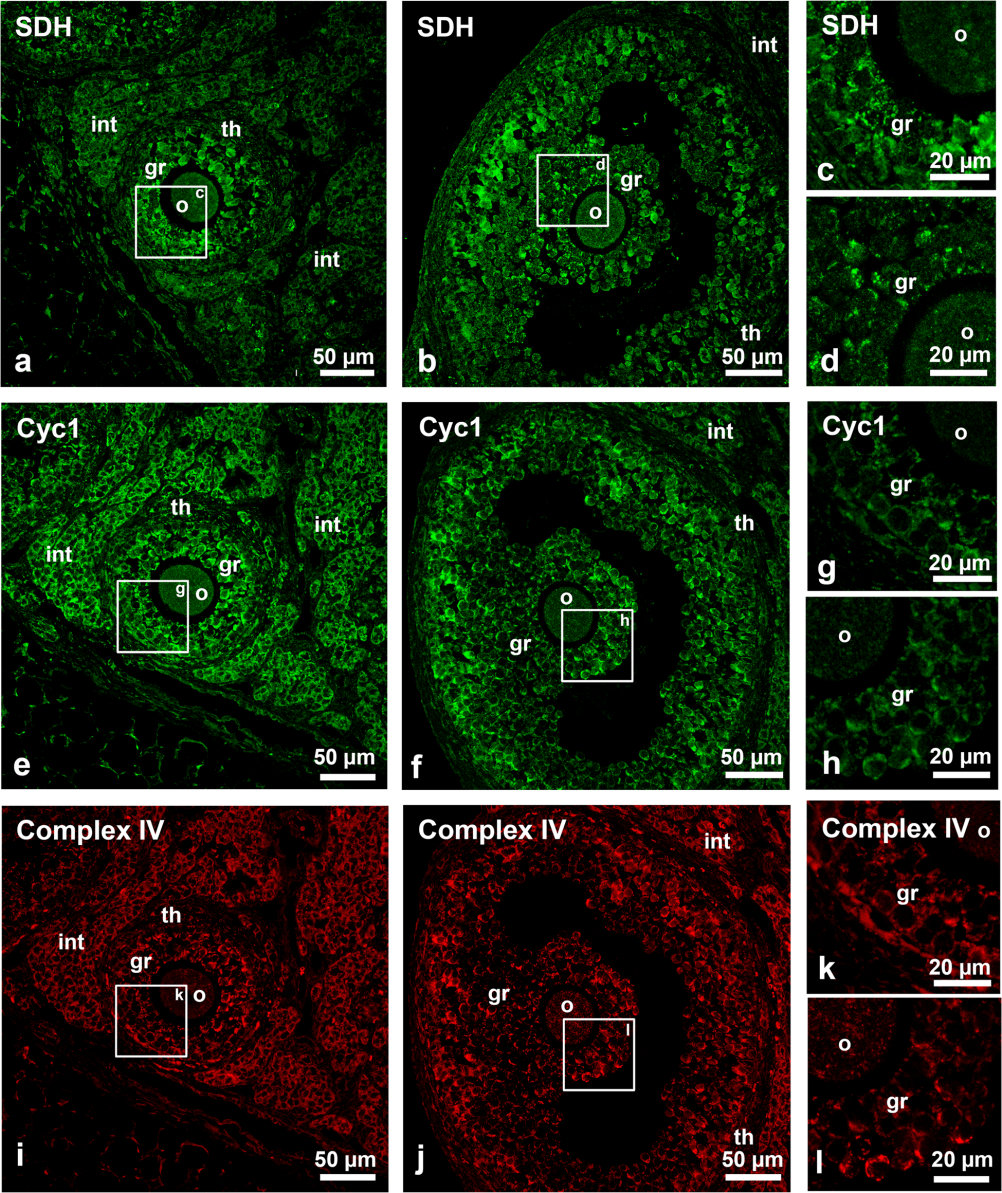
Immunofluorescence analysis of ETC complexes in secondary and tertiary follicles. **a**–**h** Ovary sections incubated with antibodies against SDH (**a**–**d**), Cyc1 (**e**–**h**) and Complex IV (**i**–**l**). in secondary (**a**, **c**, **e**, **g**, **i** and **k**) and tertiary follicles (**b**, **d**, **f**, **h**, **j** and **l**). Small images in the third image column represent magnification of the overview images of the secondary and tertiary follicles as indicated by the white squares labelled with the appropriate letter. Abbr.: o, oocyte; gr, granulosa cells; th, theca cells; int: interstitial hormone producing cells

One of the causative agents for oxidative stress in cells is the mitochondrial electron transport chain (ETC). We investigated the follicular distribution of ETC complexes using antibodies directed against succinate dehydrogenase (SDH), cytochrome c1 (complex III) and cytochrome c oxidase (complex IV, subunit 1) (Fig. 8a–l). Because of their common function and subcellular localization, the distribution of these proteins showed some similarities: (i) high abundance in granulosa cells, (ii) low abundance in oocytes and theca cells and (iii) generally uneven distribution in the granulosa cells of tertiary follicles (Fig. 8a–l). The relative staining-intensity for Cyc1 and Complex IV in the interstitial cells was stronger than the one for SDH in the same cell type (Fig. 8e–f and i–j). Double staining using antibodies directed against the two proteins demonstrated the colocalization of Cyc1 and Complex IV (Supplemental Fig S5).

## Discussion

### The abundance of peroxisomes is high in steroid hormone-producing cells and varies during the oestrus cycle

During the process of folliculogenesis, the follicle undergoes significant changes in morphology and protein composition facilitating oocyte maturation (Pedersen and Peters 1968; Hirshfield 1991; McGee and Hsueh 2000). In the ovary, ultrastructural analyses showed that in interstitial, granulosa and in granulosa lutein cells peroxisomes were located in proximity to mitochondria, smooth endoplasmic reticulum and lipid droplets (Böck 1972; Gulyas and Yuan 1977; Mendis-Handagama et al. 1998). Recent investigations employing immunofluorescence microscopy have revealed that catalase levels are elevated in theca cells and cells of the corpus luteum in murine peroxisomes. In addition, ABCD3 and PEX14 have been identified in oocytes throughout all phases of the oestrus cycle ((Grant et al. 2013), 2 doctoral theses of our laboratory (Distler 2015; Wang 2017); for an additional review see also (Wang et al. 2022)).

In the present paper, we provide a more detailed analysis of peroxisome distribution in distinct ovarian cell types throughout the oestrus cycle in mice. We showed that in all oestrus cycle stages PEX14-stained peroxisomes were most abundant in steroid hormone producing cells namely the interstitial, theca and granulosa cells, but that they were less abundant in oocytes. Cholesterol is a pivotal substrate during the process of steroid hormone synthesis, with its precursors being synthesised in part in peroxisomes. Whether all steps involved in the biosynthesis of cholesterol precursors are located within peroxisomes is still a matter of debate (Wanders and Waterham 2006). It has been hypothesized that the steps from acetyl-CoA to farnesyl pyrophosphate (e.g. the pre-squalene steps) occur inside peroxisomes (Magalhaes et al. 1997; Kovacs et al. 2002; Charles et al. 2020). Given the possible involvement of peroxisomes in cholesterol synthesis, we speculate that cells with high requirements for this precursor, such as steroid hormone-producing cells, should possess a more extensive peroxisomal compartment.

The numerical abundance of peroxisomes is well reflected by the abundance of PEX14-stained particles (Grant et al. 2013; Colasante et al. 2017, 2024) and might therefore be a mean to roughly estimate the metabolic requirement for peroxisomes. Quantification of the immunofluorescence analysis of PEX14 in follicles of different oestrus cycle phases resulted in three observations: (i) Independently of the cycle phase, the highest abundance of PEX14 was found in tertiary follicles; (ii) Primordial follicles displayed a very prominent PEX14 peak in the metestrus phase, while in primary follicles the PEX14 peak was in the oestrus phase; (iii) the PEX14 content within secondary and tertiary follicles was relatively stable throughout the oestrus cycle. The rationale behind the selective peak in PEX14 abundance in primordial and primary follicles during specific oestrus phases remains, at this point, a subject of speculation. It is plausible that a surge of the peroxisomal population is required to transit from one follicle stage to the next at certain time points during oestrus cycle. The relatively high and constant level of PEX14 throughout the oestrus cycle in tertiary follicles instead might reflect the requirement for a proliferated and stable peroxisomal population and for heightened activity of the ROS and lipid metabolism during the later phases of folliculogenesis.

In whole ovary homogenates, the activity of the ROS-scavenging enzyme catalase was most abundant in the metestrus (= shortly after ovulation) followed by oestrus (= shortly before ovulation), prooestrus (= before oestrus, start of cycle) and dioestrus (= after metoestrus, end of cycle) (Singh and Pandey 1998). Consequently, the H_2_O_2_ abundance declined in inverse manner (Singh and Pandey 1998). This suggested that catalase-mediated oxidative stress management is particularly required during ovulation and the period shortly thereafter, hence in the late phases of the oestrus cycle. The proliferation of the peroxisomal compartment in the late phases of folliculogenesis may also suggest a requirement for increased lipid β-oxidation or the synthesis of plasmalogens and steroid hormones. It is evident that further research will be necessary to explain the changes that occur in the peroxisomal compartment during the oestrus cycle, in addition to the immunofluorescence analyses presented herein. Such future investigations should include the measurement of the activity of catalase and of the peroxisomal enzymes involved in beta-oxidation and the synthesis of sterol at various oestrus phases stages.

Only very little information is available on the regulation of the peroxisomal metabolism and proliferation during folliculogenesis in response to metabolic modulators such as gonadotropic hormones or peroxisome proliferator activated receptors (PPAR). However, the latter aspect must be considered when contemplating peroxisomal functions, as intermediates of peroxisomal metabolic reactions are thought to act as ligands for PPARs (Colasante et al. 2015). Studies also proposed a relationship between the leptin receptor and PPARγ in the pathogenesis of polycystic ovary (Liang et al. 2019). Furthermore, experiments showed that in mice, PPARγ agonists exhibit a positive effect on ROS levels in polycystic ovary by enhancing the expression of catalase (Rezvanfar et al. 2012).

The activity of catalase can also be stimulated by FSH and by serum gonadotropin in granulosa cells (Behl and Pandey 2002). The abundance of catalase during follicular development was significantly increased after gonadotropin stimulation (Klinken and Stevenson 1977; Russo and Black 1982; Behl and Pandey 2002). There is also a link between the process of female steroidogenesis and peroxisomes as it has been shown that luteinizing hormone (LH) stimulation induces peroxisomal sterol carrier protein 2 (SCP2) for mitochondrial delivery of lipid droplet cholesterol (Mendis-Handagama et al. 1998), while MFP2 was suggested to catalyze the oxidation of oestradiol to estrone (Dieuaide-Noubhani et al. 1996; de Vet et al. 2002).

### Catalase is highly abundant in interstitial cells and granulosa cells but almost undetectable in oocytes under the same experimental conditions

Although ROS are crucial signaling molecules regulating various physiological processes including follicular growth, steroid synthesis and ovulation, their excess, triggered by either aging or exposure to environmental toxins, induces apoptosis and developmental defects in oocytes and embryos. Prolonged exposure of germinal tissues to ROS can onset infertility and genetic anomalies (Miyazaki et al. 1991; Agarwal et al. 2005; Shkolnik et al. 2011; Prasad et al. 2016; Pandey et al. 2018; Aitken 2020). Therefore, during follicular ripening, several antioxidative enzymes such as catalase, SOD2 and glutathione peroxidase and molecular antioxidants such as glutathione, cysteine and vitamin C are in place in the follicular cells and fluid to protect the integrity of the oocyte’s DNA and to prevent apoptosis of oocytes as well as of follicular cells (Singh and Pandey 1998; El Mouatassim et al. 1999; Behl and Pandey 2002; Cao et al. 2022).

The peroxisomal enzyme catalase possesses both (i) catalase activity: breakdown of hydrogen peroxide to water and oxygen and (ii) peroxidatic activity: metabolic breakdown of a variety of substrates, including ethanol and methanol (Fahimi 1969; Angermüller et al. 2009). The presence of catalase in whole ovary lysates and in isolated granulosa cells is relevant for follicular development as its activity increases during follicular growth and differentiation (Peterson and Stevenson 1992; Behl and Pandey 2002). Interestingly, granulosa cells of polycystic ovary patients display increased ROS levels as well as reduced catalase activity (Mazloomi et al. 2023).

Our immunofluorescence analysis confirmed that catalase is present in significant concentrations in interstitial and granulosa cells of secondary and tertiary follicles indicating an increased requirement for detoxification through catalase activity in these cells. Indeed, experiments demonstrated that exposure of granulosa cells to heat shock after catalase silencing, induced ROS accumulation, which resulted in apoptosis and cell cycle arrest. This process was accompanied by defects in the mitochondrial membrane potential and in the biosynthesis of steroid hormones (Khan et al. 2020). Granulosa cells nourish and maintain the quality of the enclosed oocyte by providing paracrine factors, such as steroid hormones, growth factors and messengers, and metabolic substrates, such as pyruvate, cholesterol, glutathione, fatty acids or amino acids, via gap junctions and membrane contact sites (Su et al. 2009). Also, mitochondria, endosomes and lysosomes as well as nucleic acids are translocated to the oocyte through specialized phylopodia, the so-called transzonal projections, that stretch across the zona pellucida from the granulosa cells (Heller et al. 1981; Albertini et al. 2001; Su et al. 2009; Doherty et al. 2021). Our results showed that in the granulosa cells electron transport chain (ETC) complexes are highly abundant, suggesting the presence of active mitochondria. We speculate that the numerous metabolic activities and transport processes of the granulosa cells increase the risk of ROS accumulation, making the presence of antioxidant enzymes such as catalase essential in these cells.

Interestingly, despite the notable presence of PEX14-positive peroxisomes in secondary and tertiary follicle oocytes, our results demonstrated that these peroxisomes exhibit strikingly low abundance of catalase. This was unexpected, given that earlier studies had suggested that catalase should act as a protective enzyme for oocytes (DeJong et al. 2007; Diaz-Albiter et al. 2011). Catalase expression in oocytes, however, is species-dependent (Harvey et al. 1995; DeJong et al. 2007; Park et al. 2016) and conflicting evidence exists in the literature regarding the presence of catalase in mouse and human oocytes (Harvey et al. 1995; El Mouatassim et al. 1999). Investigations reported that catalase-labelled peroxisomes were present in low abundance in rat oocytes, in contrast to granulosa cells (Figueroa et al. 2000). An early EM study using DAB staining on ovarian chambers of the fruit fly *Drosophila melanogaster* reported that catalase activity was present exclusively in oocytes in the very last stages of oogenesis (Giorgi and Deri 1976). In quails, catalase activity was observed by DAB staining exclusively in the small previtellogenic stages, but its activity was not detectable in any of the vitellogenic stages (Espeel et al. 1997). Furthermore, catalase transcripts were not detected either in mouse nor in human germinal vesicle and metaphase II oocytes (El Mouatassim et al. 1999). A previous hypothesis proposed that reduced catalase gene expression in vitellogenic oocytes caused the protein to become diluted within the rapidly growing population of peroxisomes (Espeel et al. 1997). The controversy surrounding the presence of catalase within oocytes is likely attributable to the utilization of disparate methodologies for its detection and the employment of distinct animal models, which renders the comparability of the results very difficult. Our study supports the hypothesis that in mouse oocytes catalase is present at negligible levels in peroxisomes. Analogously to pancreatic b and a cells (Gurgul et al. 2004), it appears that catalase is a “disallowed” gene, probably because its activity disrupts the normal function of the oocytes of mice. In which way catalase may interfere with oocyte activity remains to be elucidated and necessitates further investigation. Such research should involve the quantification of catalase activity in oocytes and granulosa cells to ascertain the extent of the effective enzymatic activity within these cells. Further experiments beyond immunofluorescence analyses could also provide insight into how mouse oocytes deal with ROS in the absence of catalase. Here, we would like to propose 3 hypotheses for consideration:

i. Delegation of ROS-producing metabolic reactions to the surrounding granulosa cells: During folliculogenesis, oocytes enter a state of dormancy that can endure for extended time periods prior to their release during ovulation (“quiet embryo” hypothesis) (Leese 2002). During quiescence, oocytes exhibit some degree of metabolic activity, linked to the biosynthesis of essential biomolecules (Leese 2002). This process, however, concomitantly introduces the potential for the accumulation of ROS over time. To circumvent the generation of ROS from metabolic reactions, it was proposed that oocytes outsource numerous metabolic pathways to neighboring granulosa cells including the provision of metabolites of energy metabolism such as pyruvate but also ATP (Su et al. 2009; Xie et al. 2023).
  In support to this hypothesis, our immunofluorescence analysis indicated that, in comparison to the surrounding granulosa cells, oocytes contain only low amounts of mitochondrial electron transport chain (ETC) complex subunits. These findings support previous research, which showed that lowering the expression and activity of mitochondrial ETC proteins, and particularly of complex I, protects the oocyte from the accumulation of ROS (Rodríguez-Nuevo et al. 2022). Since the production of ATP through the ETC complexes (mainly complexes I and III) is one of the main sources of ROS production within the mitochondria, reducing their abundance could lower ROS concentrations in the oocyte and reduce the need for catalase.
ii. Catalase is redundant due to the presence of other, more efficient enzymes that scavenge ROS within mice oocytes. Our results suggest that neither catalase nor SOD2 are the preferred antioxidative enzymes in these cells. In contrast, the abundance of GR was extremely high in the oocyte. GR plays a crucial role in recycling glutathione disulfide to maintain the intracellular pool of reduced glutathione. This, in turn, is necessary for scavenging ROS, thereby contributing to the regulation of the cellular redox state. In this respect, Tiwari and colleagues reported that cumulus granulosa cells protect the oocyte from oxidative stress (Tiwari et al. 2017), maybe by transferring glutathione from the granulosa cells to the oocyte (Shi and Sirard 2022). This could explain the high abundance of GR in the oocyte. Also, in previous experiments high GR abundance was shown by immunohistochemistry in the oocytes as well as in granulosa cells of rats (Kaneko et al. 2001). Analogously, it was postulated that glutathione peroxidase (GPx) activity, that transfers the electrons from glutathione to H_2_O_2_ and reduces it to water, is more convenient for the oocyte than the H_2_O_2_-scavenging activity of catalase (El Mouatassim et al. 1999). Probably the activity of GPx is more profitable since it is also capable of detoxifying lipid peroxides (El Mouatassim et al. 1999). The presence of GPx3 was recently demonstrated in mouse oocytes (Kreheľová et al. 2025). It is therefore not surprising that GR, required for the recycling of the oxidized glutathione, should be present in high abundance in the oocyte.
iii. ROS are transferred from the oocyte to the surrounding granulosa cells for degradation. Much is known about the molecules that are transferred from granulosa cells to oocytes. However, lately, increasing evidence indicates that some molecules, including signaling molecules and ribose 5-phosphate, are transferred from the oocyte to the granulosa cells (Martinez et al. 2023). The present study suggests that 8OHdG localizes preferentially at the TZPs in a circular fashion surrounding the oocyte. Furthermore, the inner granulosa layer directly facing the oocyte contained particularly high amounts of catalase. These observations might indicate that oxidized molecules, possibly also H_2_O_2_, are positioned in close proximity to granulosa cells so as to be transferred to and subsequently degraded by the latter by antioxidative enzymes including catalase. In this regard, it will be necessary to ascertain the source of the observed 8OHdG. We might speculate that this molecule is (i) produced in the oocyte and transferred to the edges of the oocyte; (ii) the result of ROS leaking from the granulosa cells to the oocyte through gap-junctions or (iii) located within the granulosa cells, at the distal end of the TZP communicating with the oocyte surface giving the impression of a ring-like localization at the periphery of the oocyte. Further studies will be necessary to determine the origin of the observed 8OHdG and to ascertain the possibility and mechanistic of ROS-trafficking across TZPs.

### Peroxisomes are required for the synthesis of different metabolites for oocyte and follicle development and growth

Despite the absence of catalase, peroxisomes are numerous in the oocyte, indicating the requirement for peroxisomal metabolic functions. These might comprise the production of cholesterol precursors, cholesterol side-chain cleavage for the synthesis of steroid hormones and the synthesis of plasmalogens, important vinyl-ether lipids protecting against ROS-induced membrane lipid peroxidation. Indeed, proteins of the lipid metabolism such as ABCD3, GNPAT and MFP2 were particularly abundant in the oocyte especially in the antral follicle. Similarly, these proteins were easily detected in the surrounding granulosa and theca cells. One possible reason for this finding might be that more lipids are needed for growing the oocyte and follicle membranes, and for energy metabolism during oocyte and follicle growth and maturation, as was described earlier (Shi and Sirard 2022).

Ether lipids are integral components of biological membranes regulating their fluidity and dynamics. Moreover, they act as second messengers and as antioxidants to protect other lipids from peroxidation (Brites et al. 2004; Messias et al. 2018). The synthesis of ether lipids is initiated in the peroxisomal matrix by the enzyme GNPAT, which esterifies glycerone phosphate to a long-chain acyl-CoA ester. This is followed by the formation of an alkylglycerone phosphate by the alkylglycerone phosphate synthase (AGPS), which is located exclusively inside the peroxisomal matrix. After reduction to alkyl glycerol phosphate by the alkylglycerone phosphate reductase (PexRAP) located on the peroxisomal membrane, the ether lipid biosynthesis is finalized via 3 additional enzymes in the endoplasmic reticulum (Dean and Lodhi 2017). Interestingly, female mice with a GNPAT knockout possess smaller ovaries and are sub fertile (Rodemer et al. 2003).

It is of interest that the ether-lipid class plasmalogens is of great importance and increased under hypoxic conditions and accordingly the genes involved in their synthesis are essential during hypoxia (Jain et al. 2020). Also, in the follicular fluids of patients with polycystic ovary syndrome, the abundance of plasmalogens was increased and positively correlated with oocyte quality (Zhang et al. 2024) leading to the conclusion that plasmalogens are beneficial against hypoxia, inflammation, oxidative stress and ferroptosis in the granulosa cells (Zhang et al. 2024).

Lipid metabolism and lipid storage in the late developmental stages are important for the maturation of oocytes (Aizawa et al. 2023). Indeed, inhibition of the β-oxidation in granulosa cells resulted in impairment of oocyte maturation and development (Ferguson and Leese 2006; Dunning et al. 2011; Paczkowski et al. 2013; Elis et al. 2015). However, β-oxidation is supposed to be low in the oocyte (Bradley and Swann 2019) and is probably delegated to the surrounding granulosa cells to prevent ROS. Whether this applies to mitochondrial and peroxisomal β-oxidation is not clear as the presence and extend of peroxisomal β-oxidation activity and function has not yet been investigated in oocytes and granulosa cells. Our results suggest that the oocytes contain particularly high amounts of the peroxisomal β-oxidation enzyme MFP2 (second and third step) and of the peroxisomal lipid transporter ABCD3. In peroxisomes, the β-oxidation process is organized in two different pathways that enable the oxidation and detoxification of a variety of lipid metabolic intermediates that cannot be degraded through mitochondrial β-oxidation (Van Veldhoven and Mannaerts 1999). These include very-long-chain fatty acids, long-chain dicarboxylic acids, eicosanoids, 2-methyl-branched fatty acids (pristanic acid), and C27-bile acid intermediates (Reddy and Mannaerts 1994; Hashimoto 1996; Wanders et al. 2020). Interestingly, the multifunctional protein 2 contains in addition to its β-oxidation activity (Dieuaide-Noubhani et al. 1996) also an intrinsic 17 beta-hydroxysteroid dehydrogenase and sterol transfer activity (Leenders et al. 1996). We hypothesize several possible functions for MFP2 in oocytes: (i) The synthesis of docosahexaenoic acid (Calder 2017); (ii) Provision of metabolic intermediates for plasmalogen synthesis (Hayashi and Hara 1997) and (iii) the interconversion of steroid hormones (Markus et al. 1995; Dieuaide-Noubhani et al. 1996; Leenders et al. 1996).

## Supplementary Information

Below is the link to the electronic supplementary material.

ESM1 (DOCX 11.0 MB)

## Data Availability

All data supporting the findings of this study are available within the paper and its Supplementary Information.
