# Supplementary material for: Peroxisomes in the mouse ovary and their alterations during follicular development and oocyte maturation
Source: Cell Tissue Res. 2025 Nov 23;402(3):373–94. doi: 10.1007/s00441-025-04025-6 (PMC12727866; doi:10.1007/s00441-025-04025-6)
Supplement: Supplementary file 1 — (DOCX 11.0 MB) [file 441_2025_4025_MOESM1_ESM.docx]

**Supplemental Material**

**Peroxisomes in the mouse ovary and their alterations during follicular development and oocyte maturation**

Claudia Colasante^1,*^, Eva-Maria Distler^1,*^, Shan Wang^1,3^, Philip Grant^1,2,^, Eveline Baumgart-Vogt^1^

1 Institute for Anatomy and Cell Biology, Division of Medical Cell Biology, Justus Liebig University Giessen, Aulweg 123, 35392 Giessen, Germany

2 Psychology School, Fresenius University of Applied Sciences, Marienburgstr. 6 60528 Frankfurt am Main, Germany

3 Department of Biomedical Engineering, ST415, ST Wing, The Hong Kong Polytechnic University,11 Yuk Choi Rd, Hung Hom, Hong Kong, China.

^*^Claudia Colasante and Eva-Maria Distler contributed equally to this manuscript

Corresponding author:

Prof. Dr. med. Eveline Baumgart-Vogt,

Institute for Anatomy and Cell Biology, Medical Cell Biology Aulweg 123, 35392 Giessen, Germany

[Eveline.Baumgart-Vogt@anatomie.med.uni-giessen.de](mailto:Eveline.Baumgart-Vogt@anatomie.med.uni-giessen.de)

**Supplemental Tables**

**Supplemental Table 1** List of primary antibodies used during immunofluorescence analysis

| **Primary antibody** | **Species** | **Dilution** | **Supplier** |
| --- | --- | --- | --- |
| 8-hydroxy-2'-deoxyguanosine (8OHdg) | goat | 1:200 | Abcam, ab93295 |
| ATP-binding cassette sub-family D member 3 (ABCD3) | rabbit | 1:500 | Alfred Völkl, Ruprecht Karls University Heidelberg, Germany |
| Catalase (CAT) | rabbit | 1:500 | Denis I. Crane, School of Biomol. Biophys. Sci., Griffith Univ., Nathan, Brisbane, Australia |
| Complex IV sub I | mouse | 1:500 | Invitrogen, 459600 |
| Cytochrome c1 (Cyc1) | rabbit | 1:200 | Proteintech 10242-1-AP |
| Glutathione reductase (GR) | rabbit | 1:500 | Abcam, ab16801 |
| Glyceronephosphate acyltransferase (GNPAT) | rabbit | 1:500 | Proteintech, Manchester,UK,Cat.no: 14931-1-AP |
| Multiple functional protein 2 (MFP2) | rabbit | 1:500 | Abcam, Cambridge, UK, Cat. no: ab97971 |
| Peroxisomal biogenesis factor 13 (PEX13p) | rabbit | 1:1000 | Denis I. Crane, School of Biomol. Biophys. Sci., Griffith Univ., Nathan, Brisbane, Australia |
| Peroxisomal biogenesis factor 14 (PEX14p) | rabbit | 1:2000 | Denis I. Crane, School of Biomol. Biophys. Sci., Griffith Univ., Nathan, Brisbane, Australia |
| Peroxisomal biogenesis factor 19 (PEX19p) | rabbit | 1:300 | (Colasante et al. 2017) |
| Peroxisomal biogenesis factor 3 (PEX3p) | rat | 1:100 | (Colasante et al. 2017) |
| Peroxisomal biogenesis factor 5 (PEX5p) | rabbit | 1:300 | Steven Gould, Johns Hopkins University, Dept. Biol. Chem., Baltimore, MD, USA |
| Succinate dehydrogenase (SDH) | rabbit | 1:400 | Proteintech, 14865-1-AP |
| Superoxide dismutase 2 (SOD2) | rabbit | 1:500 | Abcam, ab13533 |

**Supplemental Table 2** List of secondary antibodies used during immunofluorescence analysis

| **Secondary antibody** | **Dilution** | **Supplier** |
| --- | --- | --- |
| anti-rabbit-IgG Alexa Fluor 488 | 1:300 | Thermo Fisher, Cat. no: A21206 |
| anti-rat-IgG Alexa Fluor 488 | 1:300 | Thermo Fisher, Cat. no: A11006 |
| anti-mouse Alexa Fluor 555 | 1:400 | Molecular Probes, A31570 |
| anti-goat Alexa Fluor 594 | 1:300 | Molecular Probes, A11058 |

**Supplemental Figures**


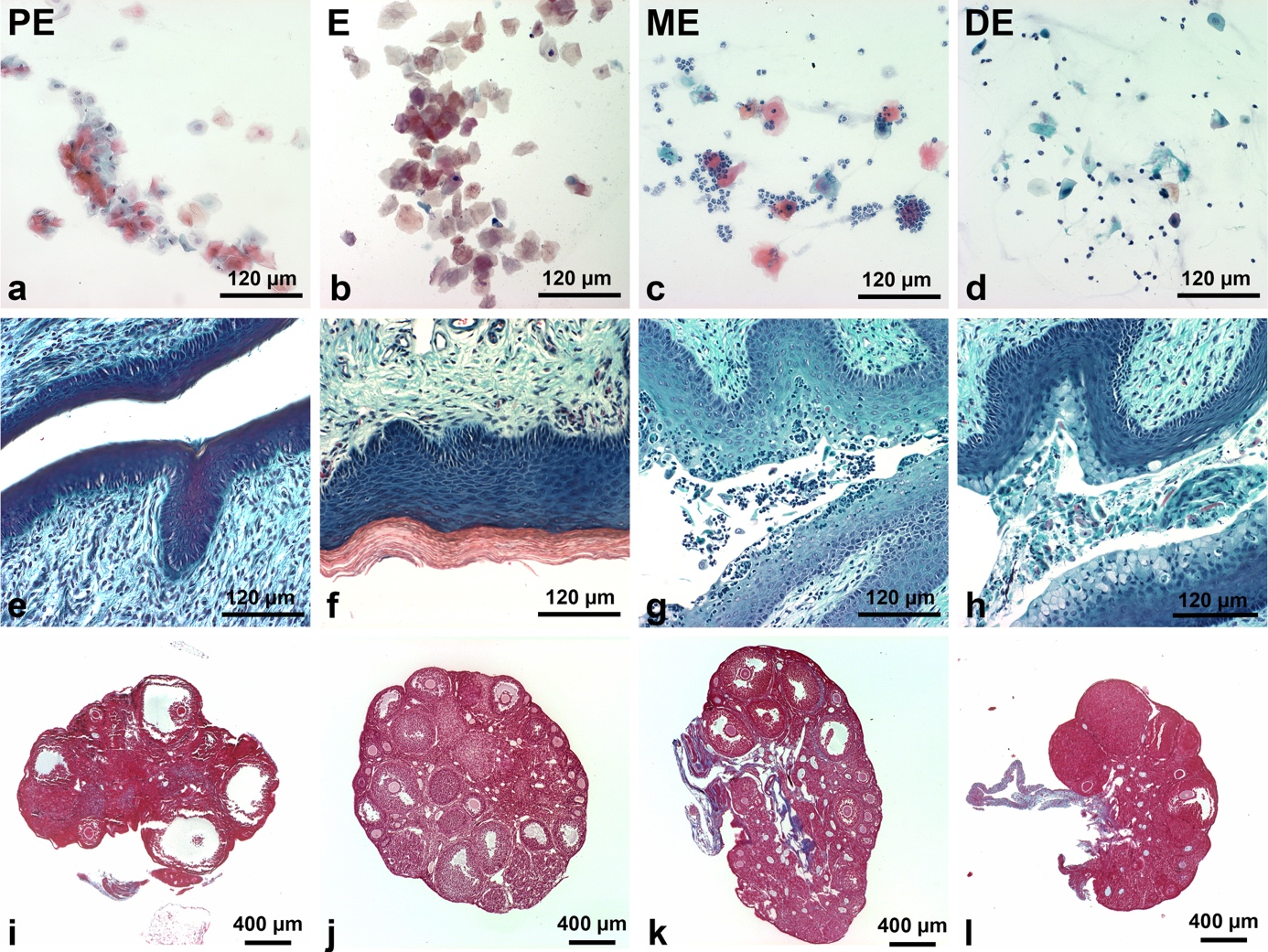


**Fig. S1 Histological determination of oestrus cycle phase.** a-d: PAP-stained vaginal smears in different oestrus cycle stages. In PE mostly large intermediate and superficial cells can be seen (a). In E the majority of cells are anuclear superficial cells (b). During the ME phase the number of neutrophylic granulocytes in the smear increases (c). In DE the superficial cells disappear and are replaced by parabasal and intermediate cells. e-h: Vaginal histology in different oestrus cycle stages assessed by PAP-staining. During PE the vaginal epithelium starts partial squamification (e) which reaches its maximum during E (f). With the onset of ME the squamified cell layer is lost and polymorphic leukocyte transmigration becomes evident (g). During DE mucus-secreting luminal cells become evident. i-l: Ovarian histology in different oestrus cycle stages assessed by Azan staining. The ovary in the PE is characterised by the presence of the corpus luteus, vacuolization and the presence of fibrous tissue (i). In the E and ME degenerated corpus luteus can be visualised (j and k). In DE a large corpus luteum is observed as well with the fibrotic tissue. PE, Proestrus; E, oestrus; ME, Metestrus; DE, Diestrus

**
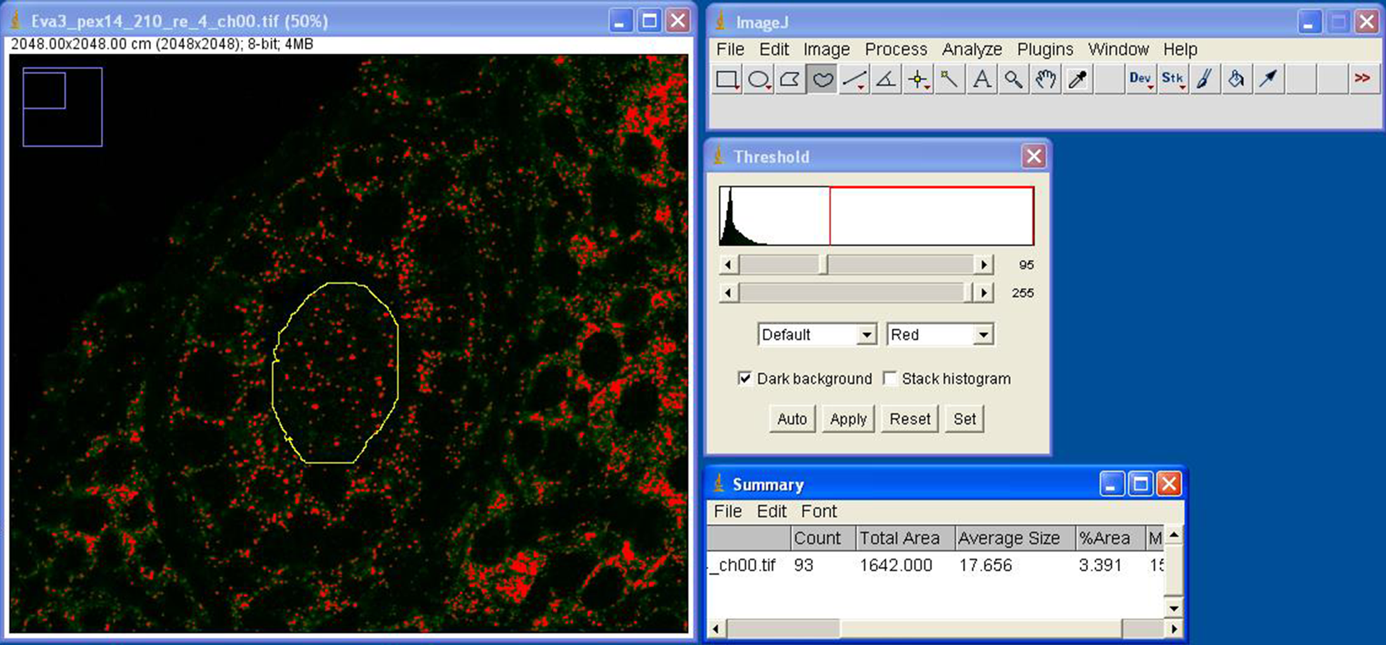
**

**Fig. S2 Selection of the oocyte region of interest (ROI) in imageJ.** After labelling of the oocyte area with a circle (see the window named “ImageJ”) and defining the threshold values (see the window named “Threshhold”) the % area of the fluorescent signals (see the window named “Summary”) was calculated by the ImageJ software program


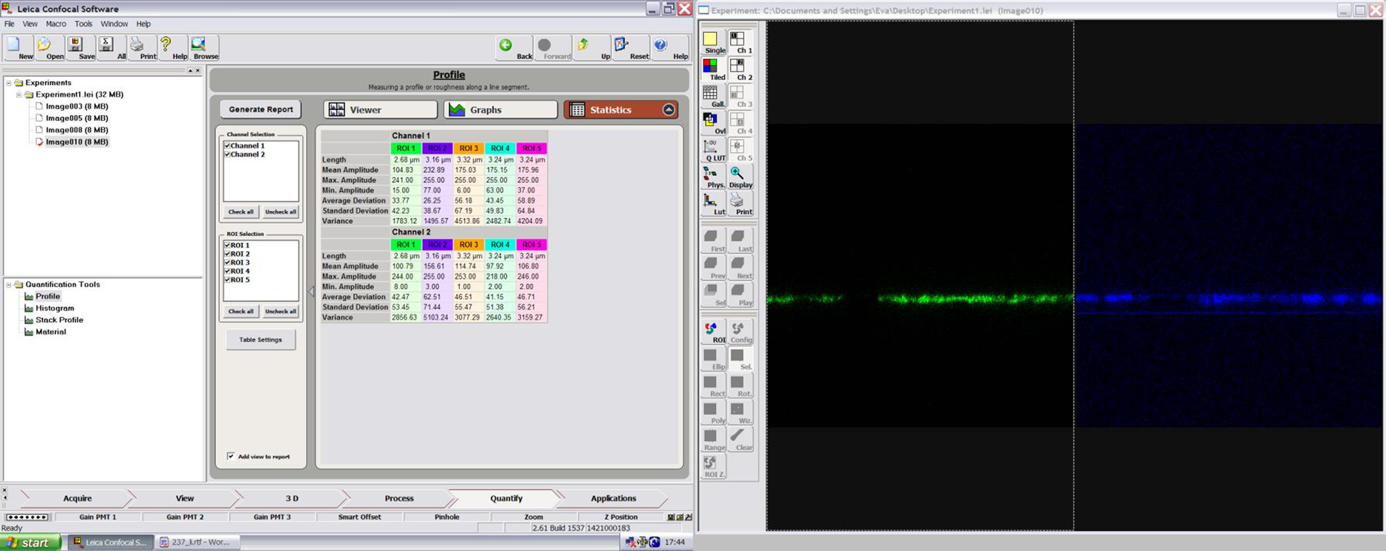


**Fig. S3 Determination of tissue section thickness.** After scanning a PEX14p labelled section in XZ-direction five different regions of interest were measured to calculate the thickness of the stained area in a paraffin section. The PEX14p labelling is shown in channel 1 in the right window in green, the nuclear staining in channel 2 in blue. The measured values at a given region of interest (ROI 1-5) are depicted in the two channels in the left window. The mean of 5 ROI values was calculated thereafter

**
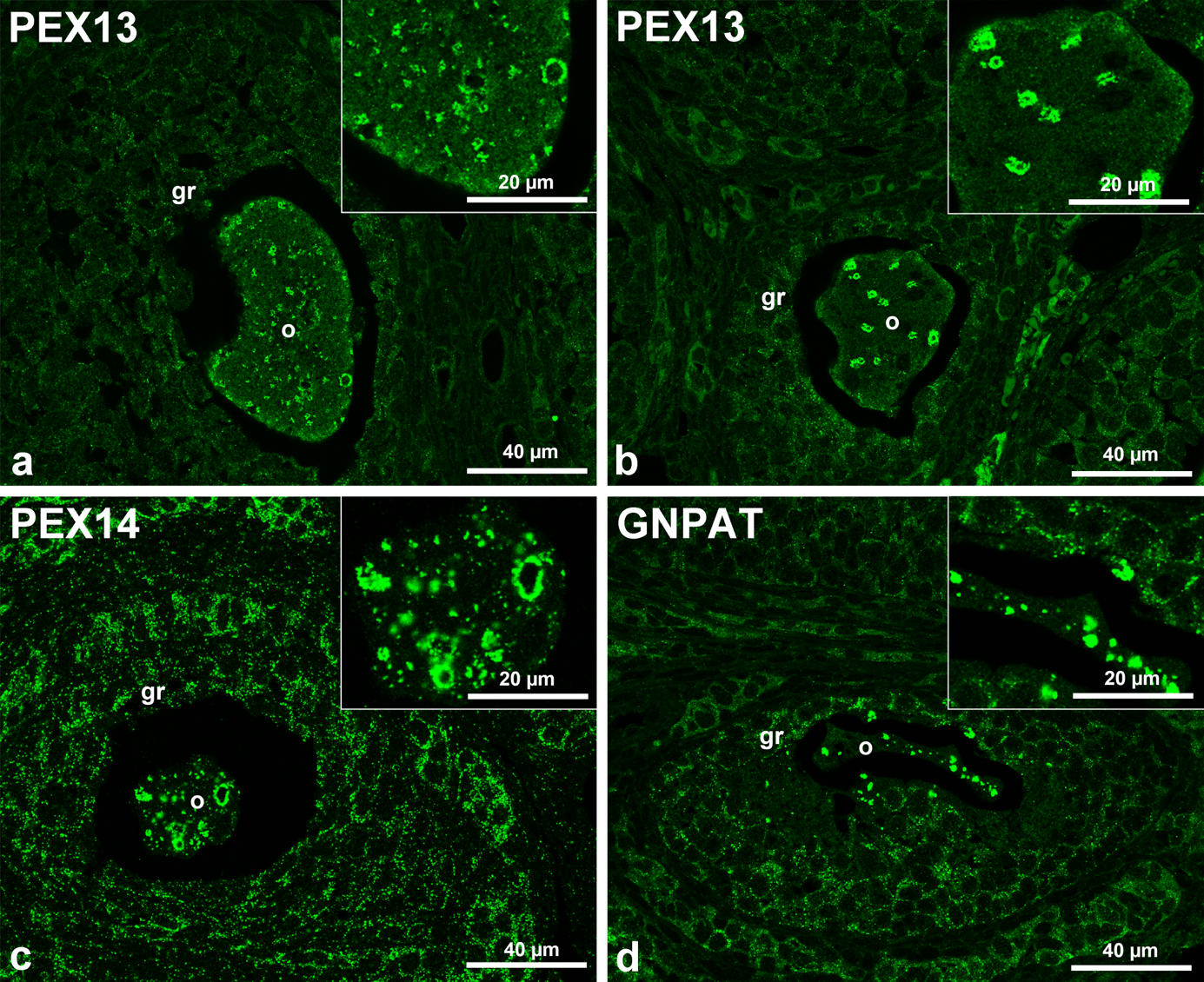
**

**Fig. S4 Immunofluorescence analysis of peroxisomal enzymes in degenerating oocytes**. a-d: The immunofluorescence analysis was performed using antibodies against PEX13p (a and b), PEX14p (c) and GNPAT (d). Abbr.: o, oocyte; gr, granulosa cells


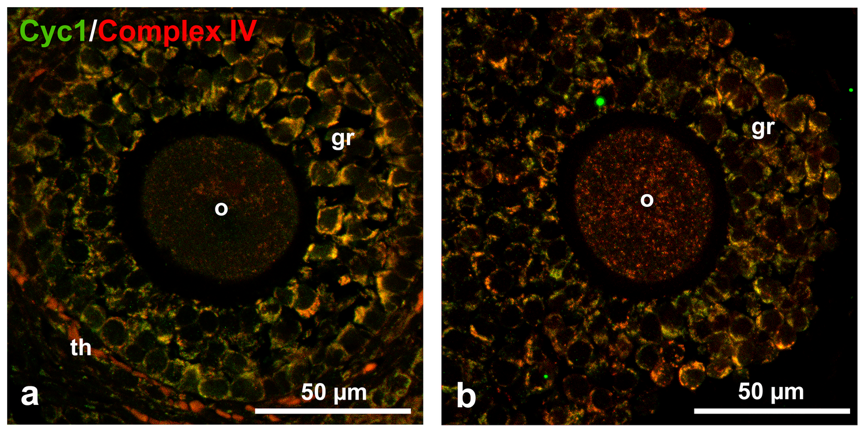


**Fig. S5 Immunofluorescence analysis of Cyc1 and Complex IV.** a-b: Colocalization of the fluorescent signal of Cyc1 (green) and Complex IV (red) from the secondary (a) and tertiary (b) follicles shown in Fig 8e, f, I and j. Abbr.: o, oocyte; gr, granulosa cells; th, theca cells
